# Fiber Bragg Sensors Embedded in Cast Aluminum Parts: Axial Strain and Temperature Response

**DOI:** 10.3390/s21051680

**Published:** 2021-03-01

**Authors:** Markus Lindner, Andrea Stadler, Georg Hamann, Bennet Fischer, Martin Jakobi, Florian Heilmeier, Constantin Bauer, Wolfram Volk, Alexander W. Koch, Johannes Roths

**Affiliations:** 1 Photonics Laboratory, Munich University of Applied Sciences, Lothstr. 34, 80335 Munich, Germany; andrea.stadler@hm.edu (A.S.); ghamann@hm.edu (G.H.); roths@hm.edu (J.R.); 2 Institut National de la Recherche Scientifique (INRS), Centre Énergie Matériaux Télécommunications, 1650 Boulevard Lionel-Boulet, Varennes, QC J3X 1S2, Canada; bennet.fischer@emt.inrs.ca; 3 Institute for Measurement Systems and Sensor Technology (MST), Technical University of Munich (TUM), Arcisstr. 21, 80333 Munich, Germany; m.jakobi@tum.de (M.J.); a.w.koch@tum.de (A.W.K.); 4 Chair of Metal Forming and Casting (UTG), Technical University of Munich (TUM), Walther-Meißner-Straße 4, 85748 Garching, Germany; florian.heilmeier@utg.de (F.H.); constantin.bauer@utg.de (C.B.); wolfram.volk@utg.de (W.V.)

**Keywords:** fiber Bragg grating, regenerated fiber Bragg grating, embedded fiber Bragg grating, casting, temperature response, strain response, tensile testing

## Abstract

In this study, the response of fiber Bragg gratings (FBGs) embedded in cast aluminum parts under thermal and mechanical load were investigated. Several types of FBGs in different types of fibers were used in order to verify general applicability. To monitor a temperature-induced strain, an embedded regenerated FBG (RFBG) in a cast part was placed in a climatic chamber and heated up to 120 ${}^{\circ }C$ within several cycles. The results show good agreement with a theoretical model, which consists of a shrink-fit model and temperature-dependent material parameters. Several cast parts with different types of FBGs were machined into tensile test specimens and tensile tests were executed. For the tensile tests, a cyclic procedure was chosen, which allowed us to distinguish between the elastic and plastic deformation of the specimen. An analytical model, which described the elastic part of the tensile test, was introduced and showed good agreement with the measurements. Embedded FBGs - integrated during the casting process - showed under all mechanical and thermal load conditions no hysteresis, a reproducible sensor response, and a high reliable operation, which is very important to create metallic smart structures and packaged fiber optic sensors for harsh environments.

## 1. Introduction

Monitoring of functional parts is of great interest and could help to improve the lifetime of these parts or to avoid failures in advance [1,2]. In the field of aerospace, embedded fiber optic sensors have been used as a real-time monitoring system in an aircraft with single-point, multi-point, or distributed sensors [3,4,5,6]. For structural health monitoring (SHM), embedded sensors are beneficial since they are permanently installed, can be positioned at critical points within the structure, and they receive protection and robustness from the structure itself. Embedding fiber optic sensors in materials has mostly been studied in the context of fiber reinforced plastics fabrication [7,8], polymer additive manufacturing [9,10], and resin potting [11]. Besides the temperature-strain decoupling and other issues, the reproducibility and hysteresis-free force transmission from the workpiece into the sensor fiber over large strain and temperature ranges represent major challenges. In addition to that, fiber optic sensors have been deployed to surveil buildings, bridges, pipelines, piles [12,13,14,15], among others. They provide a reliable, in situ, and non-destructive monitoring tool for different physical parameters such as temperature and strain.

Fiber optic sensors are getting more and more popular due to their unique advantages [16], which are e.g., a small size (typical diameter of 125 μm) [17], a wavelength multiplexing ability (multiple sensing elements in a single fiber) [17,18], their immunity to electromagnetic fields (e.g., monitor electrical power generators) [19,20,21], and their chemical resistance (suitable for harsh and high-temperature environments) [22]. Depending on the application (distributed or local sensing), there are different fiber optic sensor techniques available [23,24].

Fiber Bragg gratings (one type of fiber optic sensor) are often used in SHM applications due to their immunity to power fluctuation along the fiber, which makes them ideal for industry applications [12]. Thus, fiber Bragg gratings (FBGs) have been widely used for health monitoring of structural parts and composite materials [7,25,26]. FBGs are periodic refractive index changes inside the core of a glass fiber. Due to the periodic grating, a broadband light propagating through the fiber will be partially reflected at the FBG around a specific wavelength, called the Bragg wavelength, $\lambda_{B}=2n_{eff}\Lambda$. It is determined by the period of the refractive index modulation of the grating Λ and the effective refractive index of the fiber $n_{eff}$ [27,28,29]. If temperature, strain, force, etc. is acting on the fiber, and thus on the FBG, the Bragg wavelength will change. Both therefractive index and grating period are sensitive to temperature and strain. The refractive index changes due to the thermo-optic and elasto-optic effects if temperature or strain are acting on the FBG [30].

The refractive index modulation in a fiber core can be created in multiple ways. The most frequently employed method uses the phase mask technique in combination with a single or several UV laser light pulses of ns duration [31,32,33]. These gratings are called Type-I FBGs. However, they have the disadvantage that their refractive index modulation decays at higher temperatures [34]. Therefore, for high-temperature sensing applications, a temperature resistant type of grating is needed. One of them are the regenerated fiber Bragg gratings (RFBGs) [35]. RFBGs are Type-I FBGs written into a hydrogen-loaded fiber that has been subjected to a high-temperature treatment for several hours or days [36,37,38,39,40]. High-temperature annealing is part of the production process of RFBGs and reduces their wavelength drifts [18,41]. Another type of high-temperature stable FBGs are Type-II FBGs, which are frequently inscribed with femtosecond lasers (Type-II fs FBGs) [42]. They are inscribed either with a phase mask or with a point-by-point technique [43]. Both types have already been incorporated in various high-temperature and harsh environment sensing applications [17,18,22,44,45,46].

Embedding fiber optic sensors into metal structures has turned out to be very complex and several methods have been studied for different applications [47,48]. Most of them are based on sputtering the bare fiber with one or more layers of different metals to improve the adhesion of the fiber on the metal plates. In a second step, the metal-coated fibers were integrated into nickel or steel by an additional electroplating process or by additive manufacturing methods. These methods of embedding have several disadvantages such as the multilayer system (the additional layers of metal makes it extremely difficult to model the strain transfer from the host material to the FBG due to the different material properties, their material interactions and the thin-film characteristics of materials) and the additional manufacturing steps for the embedding process. The response of these embedding techniques to external strain or temperature has been investigated and showed a highly nonlinear behavior, large hysteresis, and delamination [9,49,50,51]. A different method to embedded FBG is ultrasonic consolidation, which has the advantage of low temperatures during the embedding process [47,52]. The embedded FBGs showed the same disadvantages as the additive manufacturing methods, such as hysteresis and delamination of the fiber and the metal [53].

In this study, we investigated optical FBG-based sensors that have been embedded in aluminum parts during the cast process without any additional metal layers. The first report on embedding FBGs during casting was given by some of the authors [54], were the strain development during the solidification process was studied. Heilmeier et al. [55] demonstrated strain measurements with embedded FBGs in aluminum parts in the plastic deformation regime. In the present investigation, tensile tests were performed at room temperature and were evaluated in the domain of elastic strains. An analytical model for the embedded sensor fiber is presented and the observed strain sensitivities are compared with theory. In a second experiment, a cast part with an embedded FBG was installed in a climatic chamber and several temperature cycles were performed and showed good agreement with an analytical model.

## 2. Theory

### 2.1. Temperature Dependence of an FBG in Casted Aluminum

In this section, an analytical model to describe an FBG embedded in cast aluminum under thermal load is introduced. Due to the high difference in the coefficient of thermal expansion (CTE) between fiber and aluminum—the CTE of aluminum is about 40 times higher than that of glass—the fiber experiences a high thermal compressive strain during cool-down. Due to the cylinder-shaped geometries of the cast part and the fiber, it can be assumed that the issue is rotationally symmetric and therefore a cylindrical coordinate system is appropriate. Inside the fiber only a homogenous pressure and no direction-dependent stress is assumed. On the outer surface of the aluminum, there is no pressure acting on it. This problem is known as *shrink-fitting* and is well known in the literature [56,57,58]. The radial stress, $\sigma_{r}$, as a function of the radius, *r* (distance from the center of the fiber), can be described by [59]:

(1) $$\sigma_{r}\left(r\right)=\left\{\begin{array}{c} \frac{a^{2}}{b^{2}-a^{2}}\left(1-\frac{b^{2}}{a^{2}}\right)p_{i},r<a\\ \frac{a^{2}}{b^{2}-a^{2}}\left(1-\frac{b^{2}}{r^{2}}\right)p_{i},r\geq a \end{array}\right.$$

**Figure 1:**
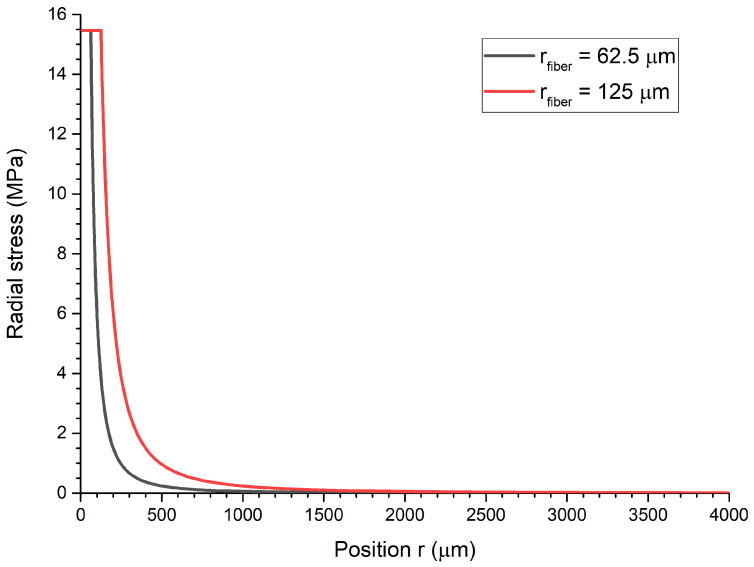
Radial stress as a function of the radial position for fibers with radii a = 62.5 μm (grey line) and a = 125 μm (red line), which are embedded in a cylindrical part made of aluminum during casting.

Here, *a* is the radius of the fiber, *b* the radius of the cast aluminum (in our case b = 4000 μm) and $p_{i}$ the internal pressure caused by the mismatch of the CTE of aluminum and glass. In Figure 1, the radial stress for two fiber radii of a = 62.5 μm (typical size of an optical fiber) and a = 125 μm (Large-mode-area fiber (LMA)) are shown. It can be seen that with a smaller fiber radius the stress inside the aluminum is stronger localized. The radial stress declines in the aluminum until it reaches zero at the boundary. Given a threshold of 1% of the maximum radial stress, the minimum aluminum radius is about a = 625 μm and a = 1250 μm, for the fiber radius of 62.5 μm and 125 μm, respectively.

Using linear elastic theory for an isotropic material, the total strains acting on the fiber can be expressed by

(2) $$\varepsilon_{r}=\varepsilon_{rr}+\varepsilon_{zr},$$

(3) $$\varepsilon_{z}=\varepsilon_{zz}+\varepsilon_{rz}.$$

The total radial strain, $\varepsilon_{r}$, is composed of the pure radial strain, $\varepsilon_{rr}$, and the transversal effect of the axial strain, $\varepsilon_{zr}$. Similarly, the total axial strain, $\varepsilon_{z}$, consists of the pure axial strain, $\varepsilon_{zz}$, and the transversal effect of the radial strain, $\varepsilon_{rz}$. Both the total radial and the total axial strain of the *shrink-fit problem* can be written as [57]

(4) $$\varepsilon_{rr}=\frac{1-\nu_{fiber}}{E_{fiber}}p_{i},$$

(5) $$\varepsilon_{rz}=-\frac{2\nu_{fiber}}{E_{fiber}}p_{i},$$

(6) $$\varepsilon_{zz}=\frac{\left(\alpha_{alu}-\alpha_{fiber}\right)E_{alu}\left(b^{2}-a^{2}\right)}{E_{fiber}a^{2}+E_{alu}\left(b^{2}-a^{2}\right)}\Delta T,$$

(7) $$\varepsilon_{zr}=-\nu_{fiber}\varepsilon_{zz}.$$

Here, $\nu_{fiber}$ is Poisson’s ratio of the fiber, $E_{fiber}$ is Young’s modulus, $\alpha_{fiber}$ the CTE of the fiber, $\alpha_{alu}$ the CTE of the aluminum and $E_{alu}$ is Young’s modulus of the aluminum. The internal pressure $p_{i}$ can be formulated as [57]

(8) $$p_{i}=\frac{A}{B-C},$$

with

(9) $$A=\left(\alpha_{alu}-\alpha_{fiber}\right)E_{alu}E_{fiber}\left(b^{2}-a^{2}\right)\Delta T,$$

(10) $$B=\left[\left(1+\nu_{alu}\right)E_{fiber}+\left(1-\nu_{fiber}\right)E_{alu}\right]b^{2},$$

(11) $$C=\left[\left(1-\nu_{alu}\right)E_{fiber}+\left(1-\nu_{fiber}\right)E_{alu}\right]a^{2}.$$

Due to the fact that the radius of the aluminum in our case is at least one order of magnitude larger than the radius if the fiber (b>>a), the Equations (6) and (8) can be simplified to

(12) $$\varepsilon_{zz}≅\left(\alpha_{alu}-\alpha_{fiber}\right)\Delta T,$$

(13) $$p_{i}≅\frac{E_{alu}E_{fiber}}{\left(1+\nu_{alu}\right)E_{fiber}+\left(1-\nu_{fiber}\right)E_{alu}}\left(\alpha_{alu}-\alpha_{fiber}\right)\Delta T.$$

**Figure 2:**
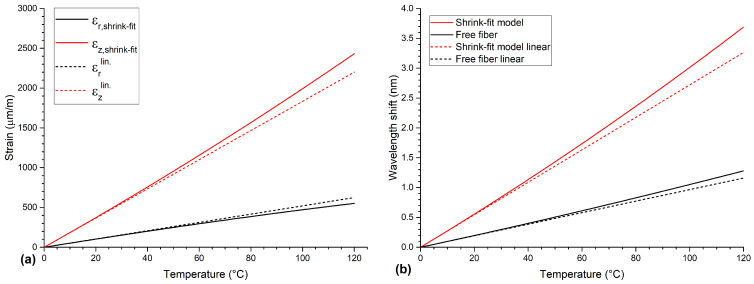
(**a**) Calculated axial (solid red line) and radial strains (solid black line) from the shrink-fit model as a function of temperature. (**b**) Wavelength shift as a function of temperature of the shrink-fit model (solid red line) and free fiber (solid black line). The wavelength shift of the free fiber is significantly lower than that of the embedded fiber. For reference, strain and wavelength shift calculations under the assumption of temperature-independent parameters (at T = 0 ${}^{\circ }$C) are depicted as dashed lines.

Note that in this case, the strain and internal pressure are independent of the radii and are dominated by the difference of the CTEs. According to Equations (6) and (8), the axial and radial strains induced by the aluminum depend nonlinearly on temperature because temperature-dependent parameters for Young’s moduli, Poisson’s ratios and thermal expansion coefficients should be used in the extended temperature range considered here. The temperature-dependent parameters are listed in Table 1. The calculated strains acting on the fiber according to the *shrink-fit model* are shown in Figure 2a as solid lines. For reference, the strains calculated with the assumption of temperature-independent parameters (at T = 0 ${}^{\circ }C$) are given in Figure 2a as dashed lines. As can be seen, when using temperature-dependent parameters the axial and radial strain are slightly nonlinear functions of the temperature. Additionally, the slope of the axial strain is increasing while the slope of the radial strain is decreasing. This increase of the axial strain is dominated by the increase of the CTE of the aluminum and the decrease of the radial strain is caused by the softening of aluminum, meaning the decrease of its Young’s modulus.

The Bragg wavelength is sensitive to strain and temperature [30],

(14) $$\lambda_{B}\left(T,\varepsilon \right)=2n_{eff}\left(T,\varepsilon \right)\Lambda \left(T,\varepsilon \right).$$

If the temperature increases, the Bragg wavelength will increase due to the increase of the refractive index (thermo-optic effect) and due to the thermal expansion of the fiber. Under an applied external strain, the Bragg wavelength will increase due to the change of the refractive index (elasto-optic effect) and the elongation of the fiber. It has been found that for a large temperature range the relationship between temperature and wavelength shift is significantly nonlinear. Hence, the temperature dependence of the Bragg wavelength should be described with a polynomial function, with polynomial coefficients $a_{i}$ as given in [17]. The change of the Bragg wavelength with strain depends on the effective elasto-optic coefficients, $p_{11}$ & $p_{12}$, and can be expressed as a function of axial ($\varepsilon_{z}$) and radial ($\varepsilon_{r}$) strains [27]. Subsequently, Equation (14) can be rewritten as,

(15) $$\Delta \lambda_{B}\left(T,\varepsilon \right)=\sum_{i=1}^{n}a_{i}\Delta T^{i}+\lambda_{B,0}\left[\varepsilon_{z}-\frac{n_{eff}^{2}}{2}\left[p_{12}\varepsilon_{z}+\left(p_{11}+p_{12}\right)\varepsilon_{r}\right]\right],$$

with $\Delta T=T-T_{0}$ and $T_{0}=0\;{}^{\circ }C$. Using Equations (4), (5), (7), (12), (13) and (15), and the mechanical parameter compiled in Table 1, the change of the Bragg wavelength as a function of temperature can be calculated and is depicted in Figure 2b as a red line.

The corresponding wavelength shift of the *shrink-fit model* is shown in Figure 2b. In addition, the wavelength shift of a free fiber (FBG without any aluminum) is shown as well and shows a lower wavelength shift compared to the *shrink-fit model*. Again, for reference, the wavelength shifts calculated with temperature-independent parameters (at T = 0 ${}^{\circ }C$) are given in Figure 2b as dashed lines.

### 2.2. FBG Embedded in Cast Aluminum during a Tensile Test

**Figure 3:**
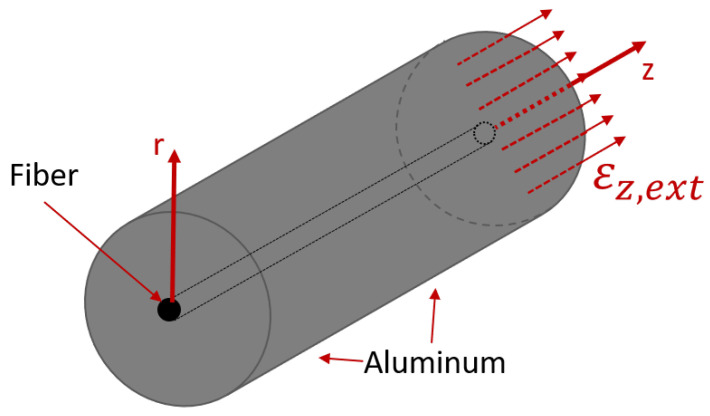
Schematic layout of the embedded fiber in aluminum with applied external axial strain, $\varepsilon_{z,ext}$, acting on both the fiber and the aluminum.

In this section, an analytical model to describe an FBG embedded in cast aluminum under external axial strain will be introduced. The model uses, as in the previous section, a cylindrical coordinate system for the fiber and the cast aluminum part due to their cylindrical geometry. This is shown schematically in Figure 3.

It is assumed that external axial strain, $\varepsilon_{z,ext}$, is acting on both the fiber, $\varepsilon_{z,fiber}$, and the aluminum, $\varepsilon_{z,alu}$, simultaneously. Additionally, the fiber and the aluminum are fixed together with no relative movement,

(16) $$\varepsilon_{z,fiber}=\varepsilon_{z,ext}\;\mathrm{and}\;\varepsilon_{z,alu}=\varepsilon_{z,ext}.$$

The total radial strains of the fiber, $\varepsilon_{r,fiber},$ and of the aluminum, $\varepsilon_{r,alu},$ can be expressed as

(17) $$\varepsilon_{r,fiber}=\varepsilon_{zr,fiber}+\varepsilon_{AF}\;\mathrm{and}\;\varepsilon_{r,alu}=\varepsilon_{zr,alu}+\varepsilon_{FA}.$$

They consist of transversal strains caused by axial strains, $\varepsilon_{zr,fiber}=-\nu_{fiber}\varepsilon_{z,ext}$, $\varepsilon_{zr,alu}=-\nu_{alu}\varepsilon_{z,ext}$, and of additional strains caused by the mismatch of Poisson’s ratios of both materials. The additional compressive strain acting on the fiber caused by the larger Poisson’s ratio of aluminum is named $\varepsilon_{AF}$ and the additional strain acting on the aluminum caused by the fiber is denoted as $\varepsilon_{FA}$. To get the unknown additional radial strains, the boundary condition at the interface of the aluminum and the fiber has to be considered. At the interface, the displacement of both materials has to be the same, which can be formulated as,

(18) $$u_{r,fiber}\left(r=a\right)=u_{r,alu}\left(r=a\right),$$

with $u_{r,fiber}$ as the displacement of the fiber and $u_{r,alu}$ as the displacement of the aluminum at radius *a*. To get the displacements, the strains have to be integrated over the fiber radius,

(19) $$\int_{0}^{a}\varepsilon_{r,fiber}dr=\int_{0}^{a}\varepsilon_{r,alu}dr,$$

(20) $$\int_{0}^{a}(\varepsilon_{AF}-\nu_{fiber}\varepsilon_{z,ext})dr=\int_{0}^{a}(\varepsilon_{FA}-\nu_{alu}\varepsilon_{z,ext})dr.$$

Due to the fact that $E_{fiber}≅E_{alu}$ it can be assumed,

(21) $$\varepsilon_{AF}≅-\varepsilon_{FA}.$$

Both integrals of Equation (20) are equal if both integrands are equal, resulting in,

(22) $$-\nu_{fiber}\varepsilon_{z,ext}-\varepsilon_{FA}=-\nu_{alu}\varepsilon_{z,ext}+\varepsilon_{FA}.$$

Here, the equation can be solved for the additional strain $\varepsilon_{FA}$ to

(23) $$\varepsilon_{FA}=\frac{1}{2}\left(\nu_{alu}-\nu_{fiber}\right)\varepsilon_{z,ext}.$$

Now, the total radial strain of the fiber can be calculated as

(24) $$\begin{array}{c} \varepsilon_{r,fiber}=\varepsilon_{zr,fiber}+\varepsilon_{AF}=\\ -\nu_{fiber}\varepsilon_{z,ext}-\frac{1}{2}\left(\nu_{alu}-\nu_{fiber}\right)\varepsilon_{z,ext}, \end{array}$$

(25) $$\varepsilon_{r,fiber}=-\frac{1}{2}\left(\nu_{alu}+\nu_{fiber}\right)\varepsilon_{z,ext}.$$

Using Equations (15) and (25) at constant temperature, the change of the Bragg wavelength due to external strain can be compiled to,

(26) $$\Delta \lambda_{B}=\lambda_{B,0}\left[\varepsilon_{z,ext}-\frac{n_{eff}^{2}}{2}\left[\left(p_{11}+p_{12}\right)\varepsilon_{r,fiber}+p_{12}\varepsilon_{z,ext}\right]\right],$$

(27) $$\Delta \lambda_{B}=\lambda_{B,0}\varepsilon_{z,ext}\left[1-\frac{n_{eff}^{2}}{2}\left[\left(p_{11}+p_{12}\right)\left(-\frac{1}{2}\left(\nu_{alu}+\nu_{fiber}\right)\right)+p_{12}\right]\right],$$

(28) $$\Delta \lambda_{B}=\lambda_{B,0}\varepsilon_{z,ext}k_{\varepsilon_{z},theory}.$$

The relative strain sensitivity $k_{\varepsilon_{z},theory}$ can be calculated with the parameters from Table 1 to

(29) $$k_{\varepsilon_{z},theory}^{smf28}=0.826.$$

Equation (27), which describes the wavelength response of an embedded FBG on external axial strain. It can be seen that for the embedded FBG, the Poisson’s ratio is substituted for the average value of the fiber’s and aluminum’s Poisson’s ratios. This results in approximately 4% higher strain sensitivities for the embedded FBG than for a free FBG ($k_{\varepsilon_{z},theory}^{smf28}=0.795$ [65]).

## 3. Experiments & Discussion

In this paper, we describe two experiments using FBGs embedded in cast aluminum addressing their response to external axial strain and temperature. Therefore, different types of FBGs, including an UV-inscribed Type-I FBG, two Type-II FBGs that were inscribed with an fs laser, and a regenerated FBG were embedded in standardized cast parts. One part with an embedded Type-I FBG and two parts with embedded Type-II fs FBGs were machined to form test specimens, which were subjected to tensile tests at room temperature. In the second experiment, a cast part containing two RFBGs in two large mode area (LMA) fibers was investigated. One RFBG was directly embedded in the aluminum and the other one was situated inside of an embedded stainless steel capillary and served as a fiberoptic temperature sensor. This instrumented cast part was exposed to temperature cycles in the range from 0 ${}^{\circ }C$ to 120 ${}^{\circ }C$.

### 3.1. Embedded FBG under Mechanical Load

**Figure 4:**
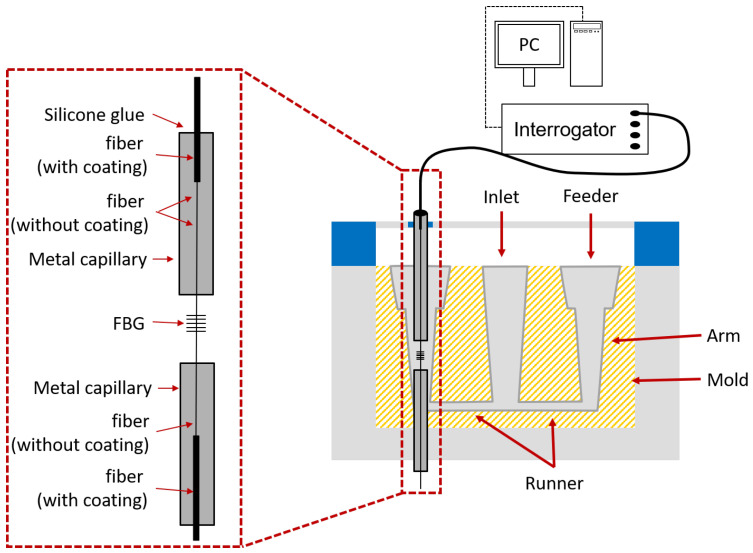
Casting setup with embedded FBG sensors. The bare fibers (smf28 or GF1B) with the FBGs get in contact with the aluminum and therefore the FBGs are sensitive to both temperature and temperature-induced strain of the aluminum.

The Type-I FBG was written into a photosensitive GF1B fiber (Nufern, East Granby, USA) with a custom in-house inscription setup, which used the phase mask technique [33]. The coating of the fiber was removed mechanically along a length of 10 cm before inscription. The Type-I FBG had a Bragg wavelength of 1550 nm and a reflectivity of 90% to 95%. The two Type-II fs FBGs were purchased from FemtoFiberTec (FemtoFiberTec GmbH, Berlin, Germany). They had reflectivities of 50% and Bragg wavelengths of 1550 nm and were inscribed into a smf28 fiber with a femtosecond laser. All three FBGs (Type-I and Type-II fs FBG) had lengths of 3 mm. Like the GF1B fiber, the coatings were removed over a length of 10 cm around the FBG. As for the GF1B fiber, in order to avoid burning the acrylate coating when exposed to the hot liquid aluminum, the coatings were removed over a length of 10 cm around the FBG. The mold geometry consisted of two identical specimens (conical geometry with a diameter of 16 mm at the bottom) connected by two runners at the bottom, a feeder at the top at each specimens, and an inlet in the middle with a filter, as illustrated in Figure 4. The runners connected the specimens with the inlet and distributed the aluminum to both specimens equally. Due to the symmetrical setup, the cast progress in both specimens was identical. Each FBG was mounted into a mold as shown in Figure 4 with metal capillaries above and below the FBG and the bare fiber with the exposed FBG (enlarged on the left side) in the middle of a specimen. The metal capillaries (outer diameter of 0.8 mm) were fixed to the frame of the mold. The metal capillaries were used to protect the fiber from unwanted shear forces and to guide the fiber. The Bragg wavelength of the FBG was sensitive to the temperature and to the strain of the aluminum as well.

**Figure 5:**
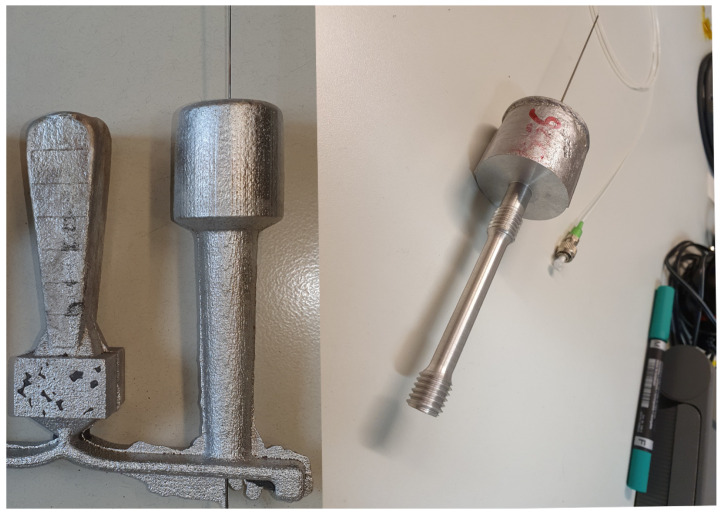
Picture of one arm of a cast part with the embedded FBG strain sensor after casting (left) and after machining (right). The casted part was machined to create a tensile test specimen according to DIN50125 with threaded ends.

The aluminum used was AlSi9Cu3 (DIN EN 1706:2010), heated up to 700 ${}^{\circ }C$ and inserted in the inlet. The aluminum flew through the runners at the bottom of the mold to both specimens and then rose upwards until it reached the top of the mold. All three FBGs survived the casting process. The Type-II fs FBGs showed an almost unchanged reflectivity after casting. Although the Type-I FBG showed a drop in grating strength to about 2% of its initial value, this was still sufficient to determine its Bragg wavelength without problems. After the cast process, each cast part with the FBG sensor was machined to a tensile test specimen according to DIN 50125. The tensile test specimen had a cylindrical geometry with a gauge length of 40 mm, a total length of 77 mm and a diameter of 8 mm with threads on each end. The threads were used to mount the specimen in the tensile testing machine. A cast part with the embedded FBG before machining is shown in Figure 5 on the left side and after machining on the right side. Afterwards, each machined specimen was mounted into a tensile testing machine (Typ 1484, ZwickRoell, Ulm, Germany). An extensometer was used to measure the axial elongation of the specimen. The attachment points of the extensometer were separated by 40 mm to each other and were located symmetrically to the position of the embedded FBG in the middle of the tensile test specimen.

The measurement procedure of the tensile tests consisted of applying and releasing axial forces in a cyclic process. The first applied force step had a magnitude of 175 N. For the subsequent steps, the force was increased by 175 N at each step resulting in force values of $F_{n}=n\times 175$ N with n as the number of the step. After each step, the force was completely released (F=0 N). The dwell times of each step, were 20 s, 60 s and 240 s respectively for the different specimens with the different FBGs. Specimen I was loaded with n = 36 cycles, Specimen II with n = 26, and Specimen III with n = 47, resulting in maximal force values of $F_{max}$ = 6.3 kN, 4.55 kN, and 8.225 kN, respectively. Different dwell times were chosen to show that the sensors perform stable under different conditions.

**Figure 6:**
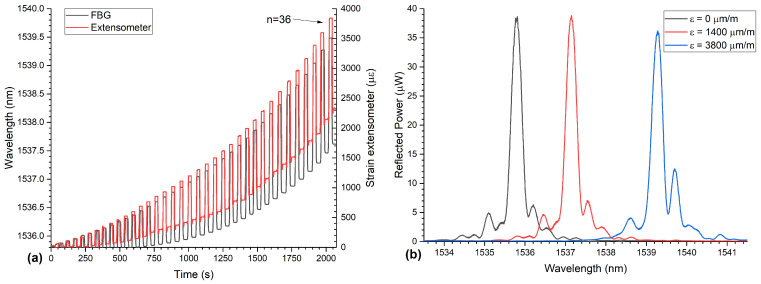
(**a**) The development of the Bragg-wavelength (here the data from the specimen with the Type-I FBG is shown as an example) and the strain as measured by the extensometer as functions of time during a tensile test experiment. The Bragg wavelengths are shown on the left axis and the strains on the right axis. The force each step was $F_{n}=n\times 175$ N, with *n* as the number of the loaded step. After each step, force was reduced to zero again. (**b**) Spectra of the FBG during tensile tests at different applied strains. The first spectrum (black line) was captured at 0 s, the second spectrum (red line) at 1160 s, and the last spectrum (blue line) at 2000 s.

The measured Bragg wavelengths and the strains measured with an extensometer as functions of time are exemplary shown in Figure 6a for the Type-I FBG as an example. In the beginning of the measurement (in the first few cycles), the strain and the wavelength values returned back to the starting values. After four cycles, the extensometer measured a plastic behavior, meaning that the strain did not return to zero (this can be seen in Figure 6a as well), but the embedded FBG sensor showed this only after 11 cycles. This indicates that the plastic strain is not distributed homogeneously along the specimen. The spectra of the FBG at three different strain levels during the tensile tests are shown in Figure 6b. During the measurement, the reflection peak of the FBG shifted towards higher wavelengths and at the last cycle (solid blue line in Figure 6b), the spectrum showed an increased side peak, which is probably caused by a strain gradient across the FBG due to the high plastic deformation of the aluminum specimen.

**Figure 7:**
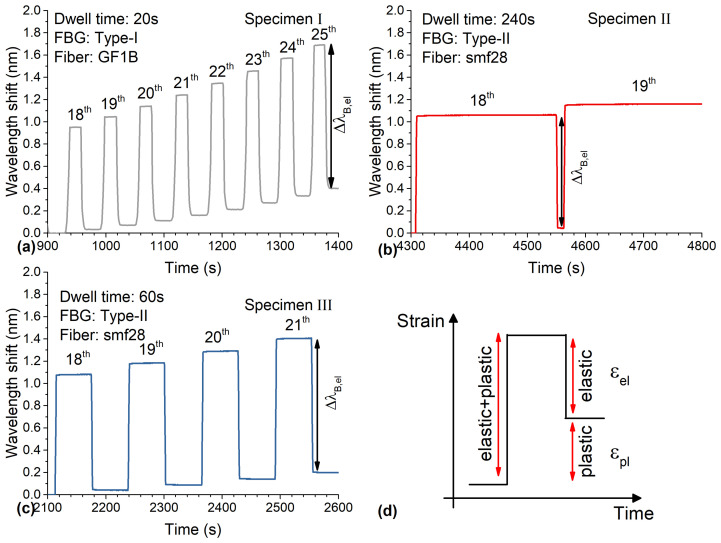
Wavelength shift of all FBGs with dwell times (**a**) 20 s, (**b**) 240 s, (**c**) 60 s after the 18th cycle, and a schematic drawing of a loading state of one cycle as a function of time. The wavelength shift of all FBGs showed an elastic and plastic behavior under force and a permanent plastic wavelength shift after the force was released. (**d**) A similar pattern was observed with the extensometer with elastic and plastic components of the measured strain (schematic representation).

**Figure 8:**
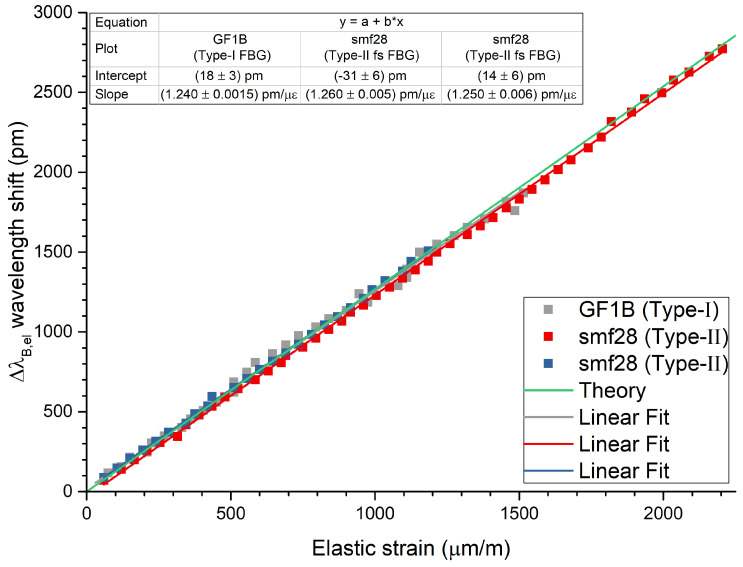
Elastic part of the wavelength shift of each FBG during tensile tests. Linear regressions (solid lines) were applied to the data points (solid squares). The theoretical wavelength shift is plotted as a solid green line as well and shows good agreement with the data.

During the tensile test experiment, the specimen experienced both plastic and elastic elongations with a total maximum strain of 3800 μm $m^{-1}$. After releasing the force, the elastic part of the strain goes back to zero and the plastic part remains. Hence, the loaded states of the specimen represent plastic and elastic strains and the unloaded states only the plastic part, which can be seen in Figure 7 in more detail. The wavelength shifts of all FBGs as a function of time at the 18th and following cycles are shown in Figure 7a–c as examples. Here, it can be seen that all FBGs follow the external applied force. The elastic strain at each cycle can be calculated by subtracting loaded from unloaded strains, $\varepsilon_{el}=\varepsilon_{loaded}-\varepsilon_{unloaded}=\varepsilon_{el+pl}-\varepsilon_{pl},$ with $\varepsilon_{loaded}$ representing the total elastic plus plastic strain and $\varepsilon_{unloaded}$ representing the total plastic strain (see Figure 7d). The corresponding wavelength shift of the embedded FBG is calculated the same way, $\Delta \lambda_{B,el}=\lambda_{B,loaded}-\lambda_{B,unloaded}$. The wavelength shifts caused by the elastic strain for all three specimens are shown in Figure 8. The data from all three specimens agree well with each other. This shows that the measurement method can be used independently from the type of the FBG. Linear functions were fitted to each of the data sets and are depicted as solid lines. The theoretical wavelength shift of the FBG as introduced in Section 2.2 is shown in Figure 8 as a green line with a slope (corresponds to the strain sensitivity) of $k_{\varepsilon }^{theory}=0.826\times 10^{-6}\frac{1}{\mu \varepsilon }1.54\mu m=1.27\;\frac{pm}{\mu \varepsilon }$. As can be seen in Figure 8, the theoretical curve and the measurements show good agreement. For comparison, the strain sensitivity of a free FBG has been reported to be $k_{\varepsilon ,free}^{theory}=1.20\frac{pm}{\mu \varepsilon }$, which is lower than the strain sensitivity of an embedded FBG.

This measurement shows that even under heavy forces with large plastic deformations, the elastic behavior of the machined part could be monitored, understood and described using an FBG. During the experiment no slip of the FBG inside the metal was present due to the large compression of the cast process, which differs from other methods such as additive manufacturing [48,66]. The slopes of the functions represent the strain sensitivities of the embedded strain sensors. The experimentally determined values of the strain sensitivities were found to be remarkably close together, which demonstrates the high level of repeatability and reproducibility of this process. The sensitivities of Specimen II and Specimen III (both with the embedded smf28 fiber and Type-II fs FBGs) differ only by ∼0.8%, which is in the range of the measurement uncertainties. Specimen I (with the embedded GF1B fiber and Type-I FBG) shows a slightly lower value (∼−1.2%) for the strain sensitivity than the average of Specimen-I and -II. This might be partly due to different dopant materials and concentrations in the cores of both types of fibers and thus to different material characteristics. A similar behavior has been found for the strain sensitivities (k-factors) of FBGs in free fibers of both types [67].

### 3.2. Embedded FBG under Thermal Load

**Figure 9:**
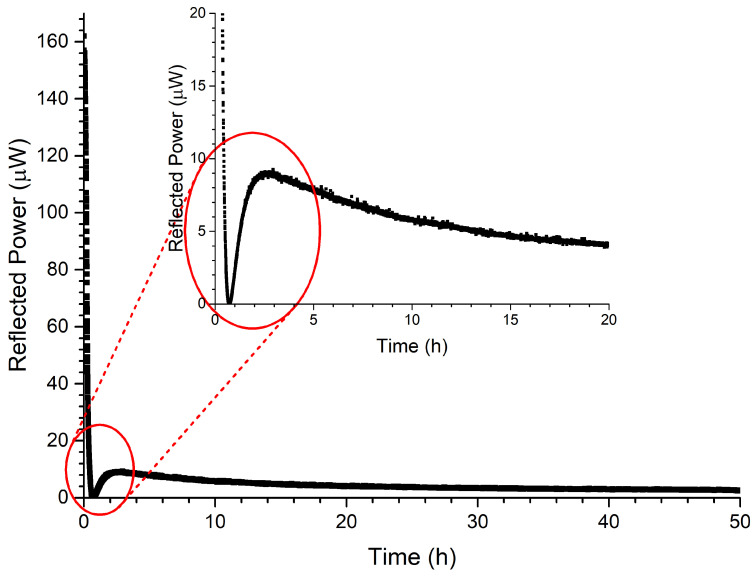
Reflected power of the FBG in the LMA fiber during the regeneration process as a function of time. During regeneration, the FBG’s reflectivity vanishes within the first hours. This is shown enlarged in the inset.

In this experiment, the influence of external temperature changes were monitored with an embedded FBG. To ensure that the FBG itself does not show any temperature-induced wavelength drift during and after the casting, RFBG sensors were used instead of Type-I or Type-II fs FBGs. A total number of two RFBG sensors, one temperature, and one strain & temperature sensor, were manufactured. Large-Mode-Area (LMA) fibers with a cladding diameter of 250 μm were used because of their increased tensile strength (4 times the cross-sectional area) compared to standard optical fibers (diameter of 125 μm). Before inscription of the Bragg seed gratings, the LMA fibers were loaded with hydrogen at 55 bar for two months at room temperature. The coatings of the LMA fibers were removed completely with acetone and standard telecommunication fibers (smf28) were spliced to each piece of LMA fiber. All seed FBGs were inscribed at Bragg wavelengths of ∼1550 nm with a grating length of 3 mm using the same custom in-house UV inscription setup mentioned above. After inscription, each piece of LMA fiber was suspended inside a high-temperature tube furnace with the FBGs positioned in the middle of the furnace. The FBGs were heated up to 900 ${}^{\circ }C$ for 48 h. During the first 15 h of this high temperature treatment, the seed FBGs transformed into RFBGs. During this time, the reflected power of the FBG decreased followed by a small recovery. This can be seen in Figure 9 with an enlarged view of the first few hours in the inset. The following 33 h were used for further annealing the RFBGs, where the reflected power decreased slowly and reached a stable value.

**Figure 10:**
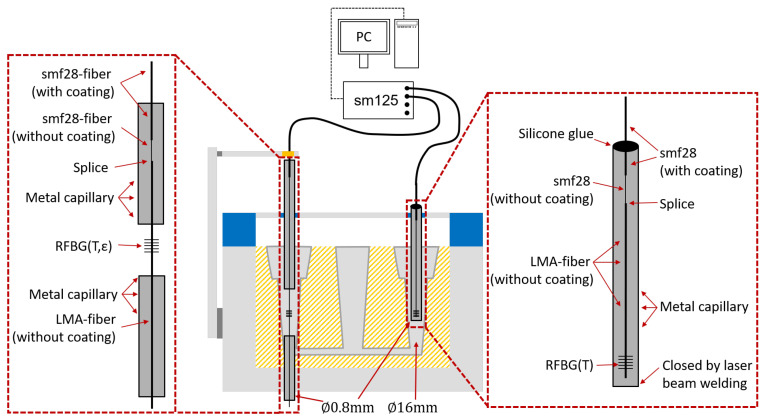
Setup for the casting process with an RFBG strain sensor (RFBG(T,ε)) and an RFBG temperature sensor RFBG(T).

The experimental setup for casting with aluminum alloy (AlSi9Cu3; DIN EN 1706:2010) is illustrated in Figure 10. The geometry of the mold was the same as described above. For fiberoptic temperature measurements, the LMA fiber was cut at 2 mm beneath the RFBG and mounted inside a metal capillary, which had an outside diameter of 0.8 mm and a length of 30 cm (see right hand side of Figure 10). One end of the metal capillary was sealed by laser beam welding. At the other end of the capillary, the fiber was fixed to the capillary with silicone glue. Due to this loose-tube packaging of the temperature sensor fiber, the RFBG was only influenced by temperature and no strain could be transferred from the aluminum onto this fiber. The second RFBG sensor was in contact with the aluminum and was installed in the other specimen of the cast part, see lefthand side of Figure 10. For ingress and egress of the fiber into and out of the aluminum, two metal capillaries were used to guide the fiber as described above. Both RFBGs were located at the same positions in each specimen of the cast part. Both fibers were connected to the interrogator (sm125, MicronOptics, Atlanta, USA), which in turn was connected to a PC for data recording. To verify that these RFBGs do not show any wavelength drift, the spectra and Bragg wavelengths were recorded during the cast process.

**Figure 11:**
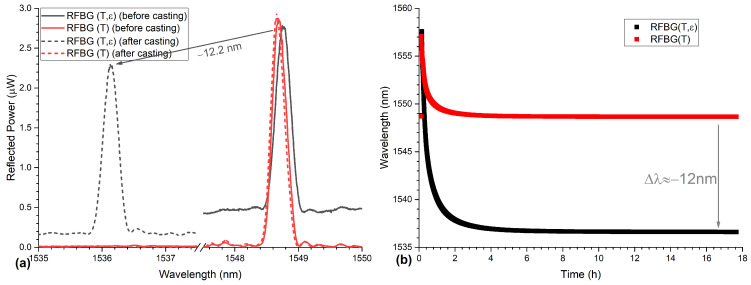
(**a**) Spectra of the RFBGs before and after casting at room temperature. The spectra of the temperature sensor (RFBG(T)) before (solid red line) and after casting (dashed red line) are superimposed, indicating no drift of the sensor occurred during the casting process. (**b**) Wavelengths of the RFBGs during the casting of the aluminum. The wavelength of both RFBGs increased at the beginning followed by the cooling of aluminum. The Wavelength of the temperature sensor went back to the starting wavelength and the strain sensor shifted ∼12.2 nm towards lower wavelengths due to thermal shrinkage of the aluminum.

The hot liquid aluminum was cast into the inlet from where the aluminum was flowing through both runners to both specimens of the cast part and raised in both specimens until it reached the top of the mold. The spectra at room temperature of both RFBGs, the strain & temperature (RFBG(T,ε)) and the temperature sensor (RFBG(T)), before and after casting are shown in Figure 11a. The spectra of the temperature sensor proves that no drift or spectral degeneration occurred during casting. The spectrum of the strain sensor shifted approximately 12.2 mm towards smaller wavelengths due to large compressive forces acting on the fiber induced by thermal shrinking of the aluminum. The wavelengths of both RFBGs as a function of time are shown in Figure 11b. At the beginning, the Bragg wavelength of both RFBGs increased when they got in contact with the hot liquid aluminum. During the cooling phase, the Bragg wavelength of the temperature sensor decreased and reached the initial wavelength when back at room temperature. The Bragg wavelength of the strain and temperature sensor reached at a smaller wavelength about 12.2 nm lower than its initial value.

**Figure 12:**
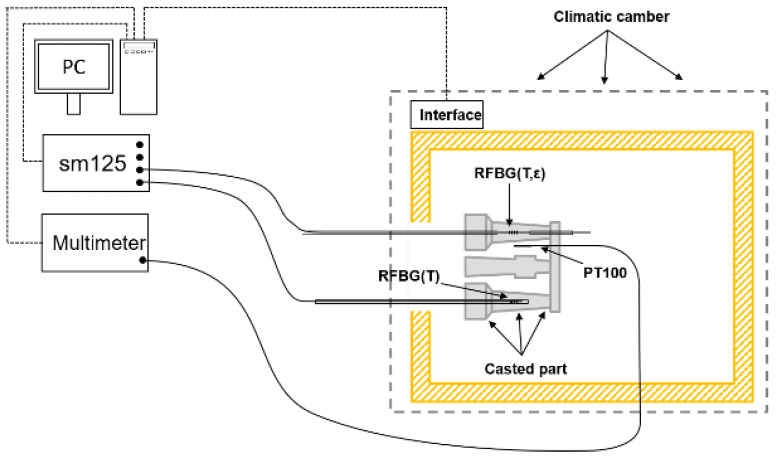
Measurement setup for the temperature characterization of embedded RFBGs in an aluminum casted part. A Pt100-temperature sensor was placed next to the casted part. The spectra of the RFBGs were recorded by a sm125 4-Channel interrogator and the resistance of the Pt100 was measured with a multimeter (Keithley2000, Tektronix GmbH, Cologne, Germany).

The casted part was installed inside a climatic chamber (VCL4010, Vötsch, Germany). The setup is shown schematically in Figure 12. A calibrated Pt100 temperature sensor was placed next to the cast part to monitor the temperature. For the measurement of the Pt100, a multimeter, and for the RFBGs a sm125 interrogator, were used. Both measurement devices were connected to a computer for data acquisition and the climatic chamber was controlled by a PC via a serial interface.

**Figure 13:**
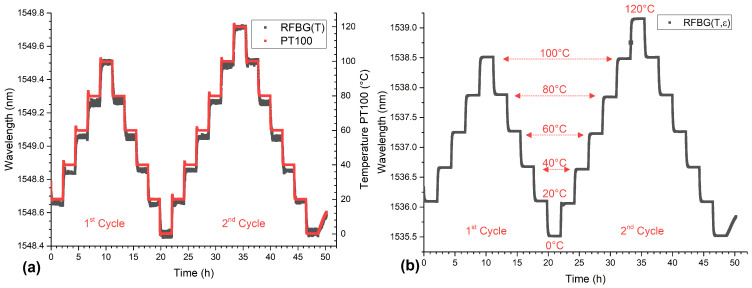
(**a**) The Bragg wavelength of the RFBG-based temperature sensor and the Pt100 temperature data as a functions of time. Both sensors followed the temperature of the chamber, the Pt100 with no and the RFBG with little delay. (**b**) The Bragg wavelengths as a function of time of the embedded strain sensor(RFBG(T,ε)). The wavelengths of the embedded strain sensor showed larger shifts compared to the temperature sensor due to thermal strain of the aluminum acting additionally on the fiber.

**Figure 14:**
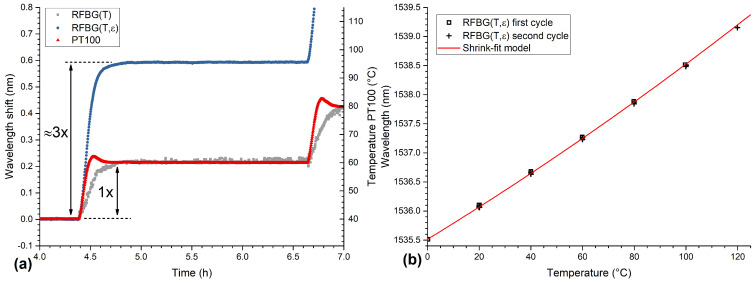
(**a**) Wavelength shift of both RFBG sensors (left axis) from 60 ${}^{\circ }C$ to 80 ${}^{\circ }C$ and the temperature from the Pt100 sensor (right axis) as a function of time. Both RFBG sensors show a larger delay than the Pt100 sensor and the embedded strain sensor has an about 3 times larger wavelength shift. (**b**) The wavelength of the embedded RFBG strain sensor (open black squares for the first cycle and crosses for the second cycle) and the theoretical model (red solid line) as functions of temperature. The analytical model agrees well with the measurements.

The temperature profile of the thermal treatments consisted of two cycles. The temperature range of the first cycle went from 0 ${}^{\circ }C$ up to 100 ${}^{\circ }C$ and the second cycle from 0 ${}^{\circ }C$ to 120 ${}^{\circ }C$ with a step size of 20 ${}^{\circ }C$ for each cycle. At each temperature step, a dwell time of 2 h was chosen so that the cast part and the climatic chamber had sufficient time to reach the thermodynamic equilibrium. During the measurement, the Bragg wavelengths of both RFBG sensors and the temperature of the Pt100 sensor were captured continuously with a measurement rate of 10 Hz. The wavelength of the RFBG temperature sensor and the temperature measured by the Pt100 sensor are shown in Figure 13a as functions of time. The Pt100 sensor followed the temperature of the climatic chamber almost instantly. The embedded RFBG temperature sensor showed a small delay due to the thermal mass of the surrounding aluminum. This can be seen more clearly in Figure 14a, where the wavelength shift from 60 ${}^{\circ }C$ to 80 ${}^{\circ }C$ is shown. The embedded RFBG strain sensor (shown as a blue circle), which was in direct contact with the aluminum, showed the same delay and a larger wavelength shift (about three times) due to the additional thermal strain of the aluminum acting on the fiber. The wavelength of the embedded strain sensor as a function of time can be seen in Figure 13b. The embedded strain sensor showed a total wavelength shift of 4.5 nm over the whole temperature range compared to the temperature sensor with a wavelength shift of 1.4 nm.

With the material parameters and the theoretical model of the embedded RFBG as introduced in Section 2.1, the expected wavelength response as a function of temperature can be calculated. This is shown in Figure 14b as a red solid line together with the measured wavelengths of both cycles (mean of the measurements during the last 60 s of each step) as a function of temperature (open black squares for the first cycle, crosses for the second cycle). Both the measured and the calculated wavelengths showed very good agreement, and the deviations between measured data and theory were found to be smaller than ±30 pm. During the measurement, almost no drift and no hysteresis have been observed. This shows that up to 120 ${}^{\circ }C$ the temperature response of embedded RFBG sensors is repeatable and can be described with a shrink-fit model and appropriate material parameters. The dependence of the temperature response of embedded RFBGs on casting parameters (alloy composition, cooling rate, etc.) and its reproducibility with different casted parts will be addressed in future research. The high compaction forces of the casted aluminum ensure that no slippage of the fiber occurs. This distinguishes the casting from other methods such as additive manufacturing and ultrasonic consolidation, which show highly nonlinear response and delamination behaviors at high temperatures [51,53].

## 4. Conclusions

The response of fiber Bragg gratings (FBGs) embedded in cast aluminum under external strain and temperature was investigated. Cast parts with embedded Type-I and Type-II fs-FBGs were machined to tensile test specimens and subsequently cyclic tensile tests were performed. The results show good agreement with the analytical solution, which was introduced in this paper. A cast part with embedded RFBG strain and temperature sensors was mounted in a climatic chamber and two temperature-cycles were carried out. Measurement results performed with the RFBG temperature sensor agreed well with results obtained from a calibrated PT100 sensor without any drift. The RFBG strain sensor showed a slightly nonlinear response and could be best described with a shrink-fit model and temperature-dependent parameters. Both experiments showed that FBGs can be used to monitor cast parts under external temperature or strain influences. In addition to that, embedding optical fibers in cast parts represents a rugged packaging solution for fiber optic sensors, which is of importance for measurements in harsh environments.

## Figures and Tables

**Table 1:** Mechanical and optical parameters ($\Delta T=T-T_{0}$, $T_{0}=0\;{}^{\circ }C$).

	Parameter	Value	Source
$E_{fiber}\left(T\right)=E_{0,fiber}+\frac{dE_{fiber}}{dT}\Delta T$	$E_{0,fiber}$	74.9 GPa	[46]
	$\frac{dE_{fiber}}{dT}$	10.2 MPa ${{}^{\circ }C}^{-1}$	[60]
$\nu_{fiber}\left(T\right)=\nu_{0,fiber}+\frac{d\nu_{fiber}}{dT}\Delta T$	$\nu_{0,fiber}$	0.16	
	$\frac{d\nu_{0,fiber}}{dT}$	$38\times 10^{-6}$ ${{}^{\circ }C}^{-1}$	
$\alpha_{fiber}\left(T\right)=A+B\Delta T+C\Delta T^{2}+D\Delta T^{3}$	A	$0.437\times 10^{-6}$ ${{}^{\circ }C}^{-1}$	Calculated from the data of [61]
	B	$2.34\times 10^{-9}$ ${{}^{\circ }C}^{-2}$	
	C	$8.9\times 10^{-12}$ ${{}^{\circ }C}^{-3}$	
	D	$1.13\times 10^{-14}$ ${{}^{\circ }C}^{-4}$	
	$n_{eff,0}$	1.4473	[62]
	$p_{11}$	0.116	[63]
	$p_{12}$	0.255	
$E_{alu}\left(T\right)=E_{0,alu}+\frac{dE_{alu}}{dT}\Delta T$	$E_{0,alu}$	78.7 GPa	Calculated from the data of [64]
	$\frac{dE_{alu}}{dT}$	−94.36 MPa ${{}^{\circ }C}^{-1}$	
	$\nu_{alu}$	0.32	
$\alpha_{alu}\left(T\right)=\alpha_{0,alu}+\frac{d\alpha_{alu}}{dT}\Delta T$	$\alpha_{0,alu}$	$22.07\times 10^{-6}$ ${{}^{\circ }C}^{-1}$	
	$\frac{d\alpha_{alu}}{dT}$	$0.01794\times 10^{-6}$ ${{}^{\circ }C}^{-2}$	
